# Sociodemographic factors and comorbidities are associated with an elevated risk of herpes simplex keratitis: a population-based study in Taiwan

**DOI:** 10.3389/fmicb.2024.1506659

**Published:** 2024-12-11

**Authors:** Ren-Long Jan, Chung-Han Ho, Jhi-Joung Wang, Han-Yi Jan, Jiun-Yi Chen, Yuh-Shin Chang

**Affiliations:** ^1^Department of Pediatrics, Chi Mei Medical Center, Liouying, Tainan, Taiwan; ^2^Department of Hospital and Health Care Administration, Chia Nan University of Pharmacy and Science, Tainan, Taiwan; ^3^Department of Medical Research, Chi Mei Medical Center, Tainan, Taiwan; ^4^School of Medicine, Tzu Chi University, Hualien, Taiwan; ^5^School of Medicine, National Yang Ming Chiao Tung University, Taipei, Taiwan; ^6^Department of Ophthalmology, Chi Mei Medical Center, Tainan, Taiwan; ^7^School of Medicine, National Sun Yat-sen University, Kaohsiung, Taiwan

**Keywords:** herpes simplex keratitis, case-controlled study, sociodemographic factors, Taiwan Longitudinal Health Insurance Database, epidemiology

## Abstract

To investigate the association among comorbidities, sociodemographic factors, and herpes simplex keratitis (HSK). This nationwide, population-based, retrospective, matched case-control study included 27,651 patients with HSK identified from the Taiwan National Health Insurance Research Database based on the International Classification of Diseases, Ninth Revision, Clinical Modification (ICD-9-CM) code 054.42 for dendritic keratitis and 054.43 for herpes simplex disciform keratitis. The age-, sex-, and index date-matched control group included 27,651 non-HSK individuals selected from the Taiwan Longitudinal Health Insurance Database 2000. Associations between HSK, sociodemographic conditions, and comorbidities were examined using univariate logistic regression analyses, and paired *t*-tests were used for continuous variables. Adjusted logistic regression was used to compare odds ratios (OR) for HSK development. Patients with corneal abrasion were more likely to develop HSK than controls [OR, 402.80; 95% confidence interval (CI), 167.47–968.79; *P* < 0.0001] even after conditional logistic regression (adjusted OR, 407.36; 95% CI, 169.35–979.89; *P* < 0.0001). Other conditions that increase the odds of HSK development include systemic diseases such as hyperlipidemia, diabetes mellitus, coronary artery disease, chronic renal disease, and human immunodeficiency virus infection. Regarding sociodemographic factors, >50% of patients with HSK were aged ≥55 years. Moreover, patients living in Northern Taiwan and metropolitan cities had higher odds of developing HSK. HSK is significantly associated with corneal abrasion, hyperlipidemia, diabetes mellitus, coronary artery disease, chronic renal disease, and human immunodeficiency virus infection.

## 1 Introduction

Herpes simplex keratitis (HSK), a viral infection affecting the cornea, is predominantly caused by the herpes simplex virus (HSV). This ocular condition is recognized for its potential to lead to recurrent episodes and visual impairment, which pose a significant clinical challenge. Patients commonly report moderate-to-severe eye pain, often described as a burning or stabbing sensation accompanied by redness and inflammation in the affected eye. Additional manifestations include tearing, discharge leading to a sticky or crusty appearance, and pronounced sensitivity to light known as photophobia. Blurred vision, foreign body sensation in the eye, and swelling of the eyelids and surrounding tissues have also been reported (Lobo et al., 2019). Corneal involvement may result in recurrent episodes of infection, marked by periods of symptom exacerbation, followed by remission. These clinical features highlight the effects of herpes keratitis on ocular health (Antony et al., 2024).

The pathophysiology of HSK involves multifaceted interactions between the virus and the host immune response, leading to inflammation, tissue damage, and potential visual impairment (Farooq and Shukla, 2012; Rowe et al., 2013). HSV enters the cornea through the epithelium, often exploiting microtrauma or breaches of the protective surface layer. Once inside the corneal epithelial cells, the virus replicates and causes local damage. The host immune response is triggered by the release of cytokines and chemokines that attract immune cells to the infected area. Inflammatory cells such as neutrophils and macrophages migrate to combat viral infections and contribute to corneal inflammation. An inflammatory response results in swelling, redness, and pain. Severe cases may lead to tissue damage, including ulceration and necrosis, compromising corneal integrity (Zhu and Viejo-Borbolla, 2021). Additionally, HSV exhibits neurotropism, establishing latency in the trigeminal ganglion with periodic reactivation that contributes to recurrent episodes of HSK. The virus employs immune evasion mechanisms, hindering major histocompatibility complex antigen presentation and interfering with interferon responses (Wang et al., 2020). If left untreated or if the immune response is inadequate, HSK can progress to complications such as stromal keratitis, which is characterized by inflammation and scarring in the corneal stroma, potentially causing vision impairment (Lobo et al., 2019).

Beyond the localized impact of HSK on the eye, a growing body of research highlights its potential association with systemic diseases. For instance, diabetes mellitus (DM) has been linked to a higher incidence of HSK because hyperglycemia can impair the corneal epithelium and enhance susceptibility to HSV infections (Kim et al., 2018; Wang et al., 2018; Rosenberg et al., 2022). Similarly, patients with human immunodeficiency virus (HIV) infection are at an increased risk of severe and recurrent HSK owing to immunosuppression (Pramod et al., 2000; Burcea et al., 2015; Sobol et al., 2016). Understanding these links is crucial for comprehensive patient care and may illuminate the broader health implications associated with HSV infections.

This study aimed to explore the relationship between sociodemographic factors and the various comorbidities associated with HSK. The investigation utilized a healthcare claims database encompassing the records of 27,651 patients with HSK and controls matched for age and sex. The primary objective of this study was to elucidate the epidemiological characteristics of HSK by analyzing this extensive dataset.

## 2 Materials and methods

### 2.1 Database

The data for this case-control study were sourced from the National Health Insurance Research Database (NHIRD) of the Taiwan National Health Research Institute. The NHIRD includes encrypted patient identification numbers and demographic information such as sex, birth date, residential area, and admission and discharge dates. It also contains International Classification of Diseases, Ninth Revision, Clinical Modification (ICD-9-CM) codes, along with records of prescriptions, diagnoses, procedures, and the costs covered by the NHI. This study was exempt from review by the Institutional Review Board of Chi Mei Medical Center.

### 2.2 Selection of patients and variables

An HSK group and a matched non-HSK control group were enrolled in this population-based case-control study. Patient information for both groups was collected from January 1, 2001, to December 31, 2013. A total of were 27,651 patients diagnosed with HSK (ICD-9-CM code 054.42: dendritic keratitis and ICD-9-CM code 054.43: herpes simplex disciform keratitis) by the NHIRD. Patients with incomplete demographic data and those diagnosed with HSK before the 1st of January 2001 were excluded.

**Figure 1:**
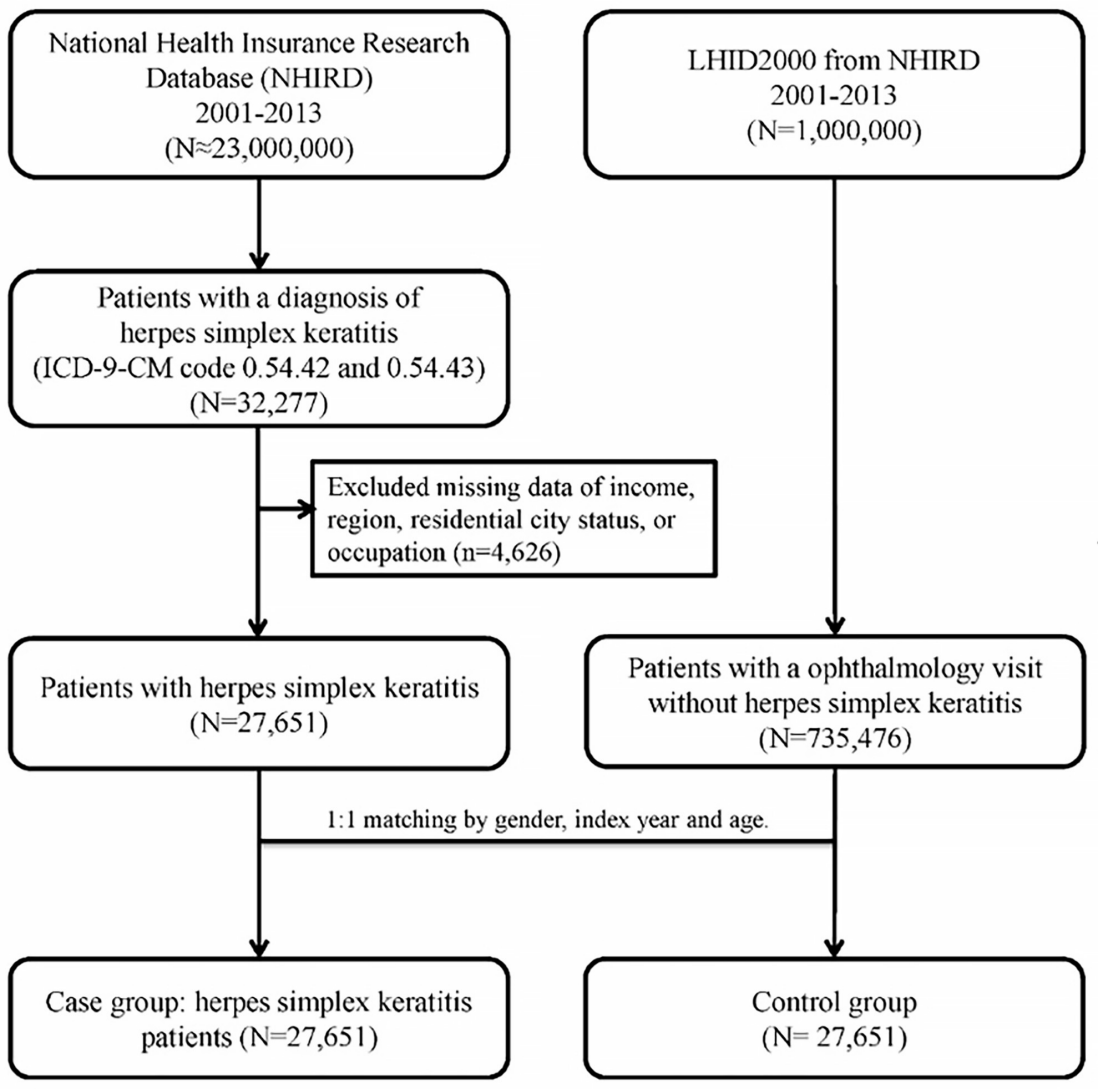
The flowchart of study subjects' selection.

For each patient with HSK, a non-HSK control was randomly selected from the Longitudinal Health Insurance Database 2000 (LHID 2000), which is a subset of patients from the larger health database and contains claims data for one million beneficiaries in 2000. The control group (*n* = 27,651) was matched to the HSK group by age, sex, and index date (defined as the first day of HSK diagnosis or diagnosis date ±30 days in ophthalmology visit for controls). Control patients with a prior diagnosis of HSK before the index date were excluded. To determine the medical comorbidities of the HSK patients, data regarding comorbid conditions such as hypertension (ICD-9-CM codes 401–405), hyperlipidemia (ICD-9-CM code 272), DM (ICD-9-CM code 250), coronary artery disease (CAD) (ICD-9-CM code 410-414), corneal abrasion (ICD-9-CM code 918.1), chronic renal disease (CRD) (ICD-9-CM code 582–588 except 584 and 587), congestive heart failure (CHF) (ICD-9-CM code 428), HIV infection (ICD-9-CM code 042 and V08), and post-organ transplantation (ICD-9-CM code 68035B, 68037B, 68047B, 75020B, and 76021B) were collected. These comorbidities were identified based on the ICD-9-CM codes recorded within the year before the index date and were ascertained using three or more ambulatory care claims or inpatient admissions. Figure 1 illustrated the selection of study subjects in a flow diagram.

### 2.3 Statistical analysis

All statistical analyses were performed using the SAS software (version 9.4; SAS Institute, Inc., Cary, North Carolina, USA). Because the HSK group was matched with the non-HSK control group, there are sufficient discordant pairs to satisfy the assumptions necessary for using McNemar's test. This test is used for estimating differences in binary categorical variables such as demographic characteristics, including sex and comorbidities like hyperlipidemia, DM, CAD, corneal abrasion, CRD, CHF, HIV infection, and post-organ transplantation. In addition, as confirmed the normal distribution using the Kolmogorov–Smirnov test (*P* > 0.05), the continuous variable, age, was assessed using paired *t*-tests to compare the difference between HSK group and controls. For categorical variables with more than two levels, such as age group, income, geographical region of Taiwan, residential city status, and occupation, Fisher's exact test was used to present the difference between cases and controls.

To estimate the association between risk factors and the outcome (HSK), odds ratios (ORs) were obtained using univariate logistic regression, and multivariate logistic regression models (conditional on age, sex, and index date) were constructed to compute the adjusted ORs for the development of HSK in patients with various comorbidities. The independent variables included sociodemographic factors (income, geographic region, residential city status, and occupation) along with all the previously mentioned medical conditions of interest. In a clinical context, a high OR value means a strong association between the risk factor and the outcome, suggesting that patients with the risk factor are at a higher risk of developing HSK compared to those without this risk factor. To address potential confounding bias, we established both a full model including all covariates and a reduced model that included only variables with a *P*-value < 0.05 from the univariate analysis to reduce the confounding effects. The fit of the logistic regression models was evaluated using the area under the receiver operating characteristic curve (AUC) to assess the discriminative ability. Statistical significance was defined as *P* < 0.05.

## 3 Results

### 3.1 Demographic data

After excluding the ineligible patients, 27,651 patients with HSK and 27,651 age- and sex-matched controls who used medical care services covered by the NHI between 2001 and 2013 were analyzed. The mean age of the patients with HSK and controls was 55.08 [standard deviation (SD) 17.34] (Table 1). The sociodemographic characteristics and comorbid conditions of the patients in the HSK and non-HSK groups are shown in Table 1. More than 50% of the patients with HSK were aged 55 years and above; 14,575 (52.71%) were men and 13,076 (47.29%) were women. The income of patients with HSK was significantly different from that of controls (*P* < 0.0001), and the most common approximate monthly income of patients with HSK was < 30,000 New Taiwan dollars (NT$) (17,123; 61.93%). With regard to geographic distribution and cities of residence, the most common region of residence of patients with HSK was Northern Taiwan (15,186; 54.92%), and the majority of the patients with HSK resided in a metropolitan city (19,819; 71.68%), which was significantly different from that of the controls (*P* < 0.0001). Regarding occupation classification, a significant difference in distribution was found between patients with HSK and controls (*P* = 0.0004), with almost half of the 27,651 patients with HSK being public servants, including military, civil, and teaching staff (13,442; 48.61%).

**Table 1:** Baseline socio-demographic factors and comorbid conditions of herpes simplex keratitis patients and age- and sex-matched control participants.

**Socio-demographic factors**	**Herpes simplex keratitis *N* = 27,651**	**Comparison *N* = 27,651**	***P* value**
	***n*** **(%)**	***n*** **(%)**	
Age (years; mean ± SD)	55.08 ± 17.34	55.08 ± 17.34	1.0000^a^
**Age (years)**			
< 25	1,261 (4.56)	1,261 (4.56)	1.0000^b^
25–34	2,855 (10.33)	2,855 (10.33)	
35–44	3,592 (12.99)	3,592 (12.99)	
45–54	5,078 (18.36)	5,078 (18.36)	
55–64	5,623 (20.34)	5,623 (20.34)	
≥65	9,242 (33.42)	9,242 (33.42)	
**Sex**			
Male	14,575 (52.71)	14,575 (52.71)	1.0000^c^
Female	13,076 (47.29)	13,076 (47.29)	
**Income**			
< NT$ 30,000	17,123 (61.93)	17,542 (63.44)	< 0.0001^b^
NT$ 30,000–60,000	8,316 (30.07)	8,139 (29.43)	
NT$ 60,000–90,000	1,619 (5.86)	1,509 (5.46)	
NT$ 90,000–120,000	320 (1.16)	251 (0.91)	
>NT$ 120,000	273 (0.99)	210 (0.76)	
**Geographical region of Taiwan**			
Northern	15,186 (54.92)	134,99 (48.82)	< 0.0001^b^
Central	4,907 (17.75)	5,528 (19.99)	
Southern	6,742 (24.38)	7,806 (28.23)	
Eastern	816 (2.95)	818 (2.96)	
**Residential city status**			
Metropolis	19,819 (71.68)	19,057 (68.92)	< 0.0001^b^
Satellite	1,586 (5.74)	1,891 (6.84)	
Rural	6,246 (22.59)	6,703 (24.24)	
**Occupation**			
Public servant	13,442 (48.61)	13,477 (48.74)	0.0004^b^
Farmer	4,512 (16.32)	4,804 (17.37)	
Fisherman	478 (1.73)	518 (1.87)	
Others	9,219 (33.34)	8,852 (32.01)	
**Comorbid conditions**			
Hypertension	8,343 (30.17)	7,640 (27.63)	< 0.0001^c^
Hyperlipidemia	4,676 (16.91)	3,621 (13.10)	< 0.0001^c^
Diabetes mellitus	4,296 (15.54)	3,508 (12.69)	< 0.0001^c^
Coronary artery disease	3,052 (11.04)	2,276 (8.23)	< 0.0001^c^
Cornea abrasion	2,015 (7.29)	6 (0.02)	< 0.0001^c^
Chronic renal disease	1,177 (4.26)	728 (2.63)	< 0.0001^c^
Congestive heart failure	643 (2.33)	607 (2.20)	0.3030^c^
Human immunodeficiency virus infection	33 (0.12)	13 (0.05)	0.0032^c^
Post-organ transplantation	7 (0.03)	5 (0.02)	0.5637^c^

NT$, New Taiwan dollars; SD, standard deviation.

^a^Paired t-test.

^b^Fisher's exact test.

^c^McNemar's test.

Patients with HSK exhibited a significantly higher prevalence of comorbid conditions such as hypertension (8,343; 30.17%; *P* < 0.0001), hyperlipidemia (4,676; 16.91%; *P* < 0.0001), DM (4,296; 15.54%; *P* < 0.0001), CAD (3,052; 11.04%; *P* < 0.0001), corneal abrasion (2,015; 7.29%; *P* < 0.0001), and HIV infection (33; 0.12%; *P* = 0.0032) than controls (Table 1).

### 3.2 Associated risk factors

Sociodemographic factors, such as income, geographic region, residential city status, and occupation, of patients with HSK and controls were evaluated using univariate logistic regression analyses and a multiple logistic regression model, adjusting for sociodemographic factors and comorbidities (Table 2). After adjusting for other confounders, patients with a monthly income greater than NT$ 30,000 had higher odds of developing HSK compared to those with an income below NT$ 30,000 (Table 2). Regarding the geographic location, odds of HSK was significantly higher in Northern Taiwan compared to that in Eastern Taiwan after a conditional logistic regression analysis (adjusted OR, 1.12; 95% CI, 1.00–1.26; *P* = 0.0463), and patients who lived in satellite cities had significantly lower odds of developing HSK when compared to those who lived in a metropolitan city, after conditional logistic regression analysis (adjusted OR, 0.78; 95% CI, 0.72–0.83; *P* < 0.0001). Patients whose occupation was public servants including military, civil, or teaching staff had a significantly lower odds of HSK compared to others after conditional logistic regression analysis (adjusted OR, 0.91; 95% CI, 0.87–0.96; *P* < 0.0001; Table 2).

**Table 2:** Odds ratios and adjusted odds ratios of various socio-demographic factors and comorbid conditions for herpes simplex keratitis.

	**Crude odds ratio^a^ (95% CI)**	***P* value**	**Adjusted odds ratio for full model^b^ (95% CI)**	***P* value**	**Adjusted odds ratio for reduced model^c^ (95% CI)**	***P* value**
**Socio-demographic factors**						
**Income**						
< NT$ 30,000	1.00		1.00		1.00	
NT$ 30,000–60,000	1.06 (1.02–1.11)	0.0030	1.06 (1.01–1.11)	0.0113	1.07 (1.02–1.12)	0.0051
NT$ 60,000–90,000	1.12 (1.04–1.21)	0.0027	1.13 (1.04–1.23)	0.0032	1.14 (1.05–1.24)	0.0021
NT$ 90,000–120,000	1.34 (1.13–1.58)	0.0007	1.35 (1.13–1.61)	0.0009	1.36 (1.14–1.62)	0.0008
>NT$ 120,000	1.36 (1.14–1.64)	0.0009	1.40 (1.16–1.70)	0.0006	1.40 (1.15–1.70)	0.0007
**Geographical region of Taiwan**						
Northern	1.14 (1.03–1.26)	0.0126	1.12 (1.00–1.26)	0.0463	1.21 (1.08–1.36)	0.0014
Central	0.89 (0.80–0.99)	0.0337	0.89 (0.80–1.00)	0.0499	0.91 (0.81–1.02)	0.1067
Southern	0.87 (0.78–0.96)	0.0055	0.86 (0.77–0.96)	0.0072	0.89 (0.79–1.00)	0.0425
Eastern	1.00		1.00		1.00	
**Residential city status**						
Metropolis	1.00		1.00		1.00	
Satellite	0.81 (0.75–0.87)	< 0.0001	0.78 (0.72–0.83)	< 0.0001	0.76 (0.70–0.82)	< 0.0001
Rural	0.89 (0.86–0.93)	< 0.0001	1.01 (0.96–1.06)	0.7863	1.02 (0.96–1.07)	0.5384
**Occupation**						
Public servant	0.96 (0.92–1.00)	0.0403	0.91 (0.87–0.96)	< 0.0001	0.92 (0.88–0.96)	0.0003
Farmer	0.89 (0.85–0.94)	< 0.0001	0.99 (0.93–1.05)	0.7558	1.00 (0.94–1.06)	0.9278
Fisherman	0.89 (0.78–1.01)	0.0621	1.01 (0.89–1.16)	0.8363	1.00 (0.88–1.15)	0.9720
Others	1.00		1.00.		1.00	
**Comorbid conditions**						
Hypertension	1.17 (1.12–1.22)	< 0.0001	1.03 (0.98–1.07)	0.2921	1.03 (0.98–1.08)	0.2794
Hyperlipidemia	1.38 (1.31–1.45)	< 0.0001	1.24 (1.18–1.31)	< 0.0001	1.24 (1.17–1.31)	< 0.0001
Diabetes mellitus	1.28 (1.22–1.35)	< 0.0001	1.13 (1.07–1.20)	< 0.0001	1.13 (1.07–1.20)	< 0.0001
Coronary artery disease	1.42 (1.34–1.51)	< 0.0001	1.30 (1.22–1.39)	< 0.0001	1.29 (1.21–1.37)	< 0.0001
Cornea abrasion	402.80 (167.47–968.79)	< 0.0001	407.36 (169.35–979.89)	< 0.0001	407.65 (169.46–980.60)	< 0.0001
Chronic renal disease	1.67 (1.51–1.83)	< 0.0001	1.54 (1.39–1.70)	< 0.0001	1.53 (1.38–1.69)	< 0.0001
Congestive heart failure	1.06 (0.95–1.19)	0.2947	0.88 (0.77–0.99)	0.0351		
Human immunodeficiency virus infection	2.54 (1.34–4.82)	0.0044	2.60 (1.36–4.97)	0.0038	2.66 (1.39–5.08)	0.0031
Post-organ transplantation	1.40 (0.44–4.41)	0.5655	1.12 (0.34–3.70)	0.8489		

^a^Crude odds ratios were obtained from univariate logistic regression analyses.

^b^Adjusted odds ratios for full model were calculated from a multivariable logistic regression model that was conditioned on age group, sex, and year of index date.

^c^Adjusted odds ratios for reduced model were included only variables with a p-value < 0.05 from the univariate logistic regression.

NT$, New Taiwan dollars; CI, confidence interval.

**Figure 2:**
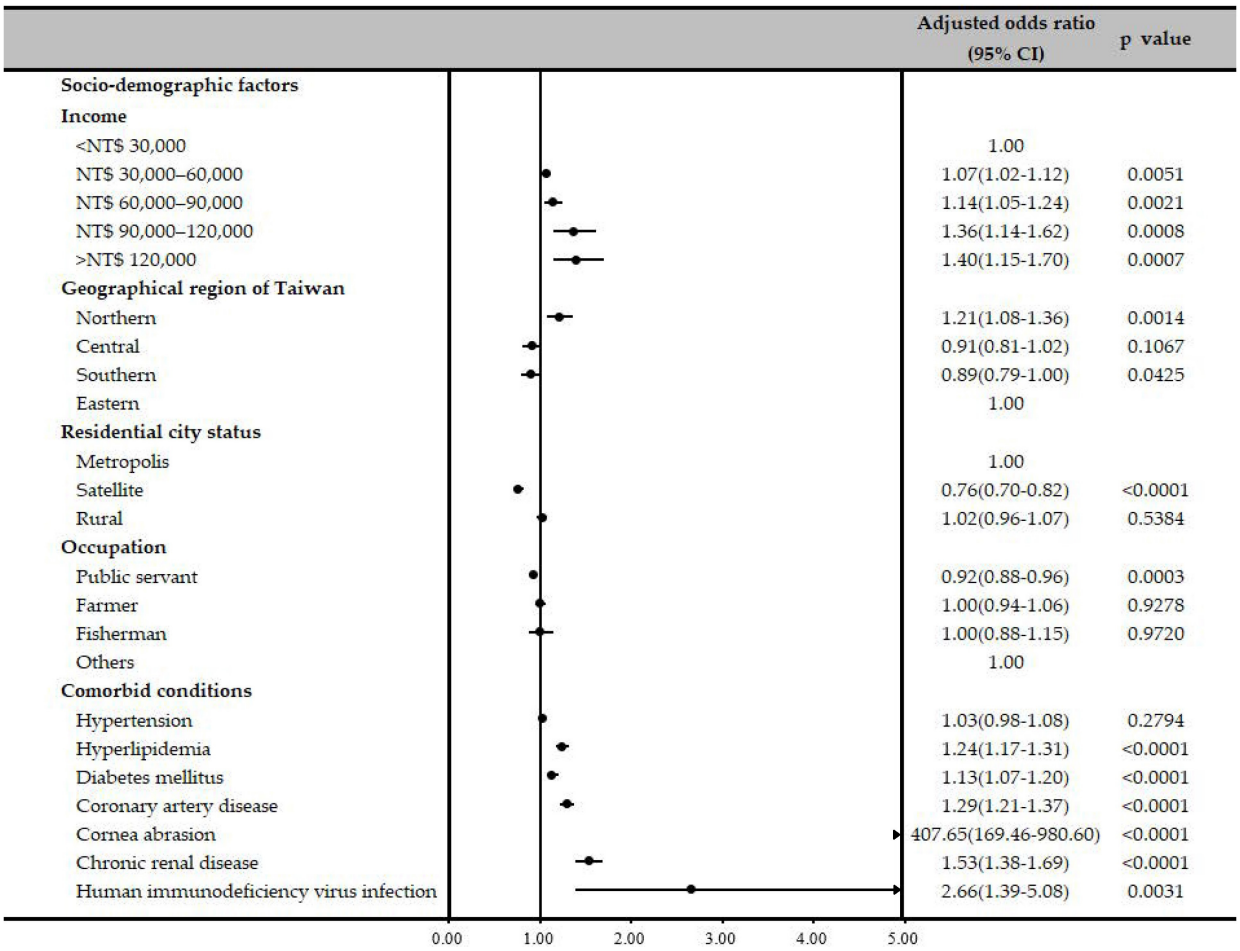
Forest plot of the adjusted odds ratios for the reduced model.

Associations between several possible comorbidities and HSK were examined using univariate and multiple logistic regression analyses (Table 2). Patients with corneal abrasion had significantly higher ORs of receiving a diagnosis of HSK (OR, 402.80; 95% CI, 167.47–968.79; *P* < 0.0001), and the OR remained high even after conditional logistic regression (adjusted OR, 407.36; 95% CI, 169.35–979.89, *P* < 0.0001). Patients with hyperlipidemia, DM, CAD, CRD and HIV infection had higher ORs of receiving a HSK diagnosis before (OR, 1.38; 95% CI, 1.31–1.45, *P* < 0.0001; OR = 1.28, 95% CI = 1.22–1.35, *P* < 0.0001; OR = 1.42, 95% CI = 1.34–1.51, *P* < 0.0001; OR = 1.67, 95% CI = 1.51–1.83, *P* < 0.0001; OR = 2.54, 95% CI = 1.34–4.82, *P* = 0.0044, respectively) and after adjustment for other confounders (adjusted OR = 1.24, 95% CI = 1.18–1.31, *P* < 0.0001; adjusted OR = 1.13, 95% CI = 1.07–1.20, *P* < 0.0001; adjusted OR = 1.30, 95% CI = 1.22–1.39, *P* < 0.0001; adjusted OR = 1.54, 95% CI = 1.39–1.70, *P* < 0.0001; adjusted OR = 2.60, 95% CI = 1.36–4.97, *P* = 0.0038, respectively). The reduced model also presented the similar results (Table 2, Figure 2). Additionally, the model's predictive accuracy for HSK was AUC = 0.5940.

## 4 Discussion

To our knowledge, this is the largest nationwide population-based case-control study to examine the relationship between sociodemographic factors, common comorbidities, and HSK. Our analysis revealed several important findings. First, over 50% of HSK patients in Taiwan were aged ≥55 years, and HSK was slightly more prevalent in men (52.71%). Second, patients residing in Northern Taiwan and metropolitan areas had higher odds of developing HSK. Third, the presence of certain comorbidities significantly impacted the likelihood of developing HSK, with patients experiencing corneal abrasion having notably increased odds (adjusted OR: 407.36; 95% CI: 169.35–979.89; *P* < 0.0001, Table 2).

Of the 27,651 patients with HSK included in this study, 14,875 (53.76%) were aged ≥55 years. This finding is consistent with those of several previous studies that have discussed the epidemiology of HSV infections (Kim et al., 2018; Grubesic et al., 2024). The higher prevalence of herpetic keratitis in individuals aged >55 years may be attributed to age-related changes in the immune system, which can lead to decreased immune surveillance and increased susceptibility to HSV reactivation. Elderly individuals often experience immunosenescence, a gradual decline in immune function that can impair the ability of the body to suppress latent HSV infections. This susceptibility is further compounded by comorbidities and potential immunosuppressive medications commonly used in older adults (Liesegang, 2001).

Regarding sociodemographic factors, we found statistically significant associations between HSK and patients living in Northern Taiwan compared with those living in Eastern Taiwan (adjusted OR, 1.12; 95% CI, 1.00–1.26; *P* = 0.0463, Table 2), particularly in metropolis cities when contrasted with satellite cities. Additionally, individuals with an income greater than NT$30,000 per month exhibited significantly higher odds of developing HSK. The elevated incidence of HSK in Northern Taiwan and the metropolitan cities in our study likely stems from several factors. These include the accessibility of affordable medical care, convenient access to ophthalmologists, and improved availability of corneal specialists for diagnosing and managing HSK relative to other regions of Taiwan. In addition, people who live in Northern or metropolitan cities and with higher incomes may suffer from more stress, leading to vulnerability to reactivation of HSV. Finally, these individuals may have greater knowledge and awareness of HSK and would immediately and actively seek help if ophthalmic problems occur.

In the present study, patients diagnosed with corneal abrasion exhibited a notably elevated OR for the development of HSK (adjusted OR, 407.36; 95% CI, 169.35–979.89, *P* < 0.0001). There is a potential link between HSK and corneal abrasion, primarily through inflammation, recurrent scarring, and neurotrophic keratopathy. HSK triggers an inflammatory cascade that helps clear the virus and contributes to progressive corneal damage, including opacification and nerve loss (Rowe et al., 2013; Lobo et al., 2019). Recurrent HSK is associated with corneal scarring and neovascularization, which can lead to visual impairment (Rowe et al., 2013; Lobo et al., 2019). HSK can lead to neurotrophic keratopathy, a condition characterized by reduced corneal sensitivity and poor healing, which may predispose the cornea to abrasion (Lobo et al., 2019).

Patients with hyperlipidemia had a remarkably higher OR for HSK development (adjusted OR = 1.24, 95% CI = 1.18–1.31, *P* < 0.0001). We reviewed numerous articles and found scant discussion on the relationship between HSK and hyperlipidemia, with only one case report addressing this topic (Amvros'eva et al., 1994). They examined the serum lipid profiles of patients with HSK and found that the severity of these lipid changes correlated with the severity of infection, with more pronounced dyslipidemia observed in recurrent cases. A direct link between HSK and hyperlipidemia has not been well documented. However, recent research has suggested a significant link between HSV infection and hyperlipidemia. Studies have found that HSV infection is associated with a higher prevalence of dyslipidemia, as evidenced by increased HSV immunoglobulin G seropositivity among affected individuals (Sun et al., 2003). Experimental models have demonstrated that HSV infection disrupts lipid metabolism, resulting in hyperlipidemia (Amvros'eva et al., 1992). This connection underscores the potential importance of managing HSK to control hyperlipidemia.

DM was determined to be an independent risk factor for HSK after adjusting for other confounders (adjusted OR, 1.13; 95% CI, 1.07–1.20; *P* < 0.001; Table 2). This finding is consistent with previous studies (Kim et al., 2018; Wang et al., 2018; Rosenberg et al., 2022). Wang et al. (2018) conducted a study compare the risk of infectious keratopathy, including HSK, in patients with or without DM and found statistically significant differences in the incidence of HSK between the two groups (*P* < 0.05). Rosenberg et al. (2022), reported that DM may be a risk factor for poor outcomes in HSK and is specifically associated with visually significant corneal scarring. Diabetes-related factors such as corneal epithelial dysfunction, chronic inflammation, and diabetic neuropathy may lead to increased susceptibility to HSK and more severe disease manifestations. The association between DM and HSK collectively underscores the critical need for the careful management of DM to mitigate its effects on HSK.

In this study, CAD was observed to be another significant risk factor for HSK (adjusted OR = 1.30, 95% CI = 1.22–1.39, *P* < 0.0001, Table 2). This study is consistent with a small number of existing reports (Kim et al., 2018), but there is limited literature specifically addressing the link between HSK and CAD. One possible explanation for this association is their shared association with chronic inflammation. Numerous studies have shown that HSV infections, including HSK, are associated with systemic inflammation (Kwon et al., 2018; Lobo et al., 2019; Musa et al., 2024). Systemic inflammation from viral infections, including HSV, has been shown to affect cardiovascular health, and studies have indicated that chronic viral infection can elevate inflammatory markers, which are known risk factors for CAD (Georges et al., 2003; Mundkur et al., 2012). These studies suggest a potential link between CAD and HSK; however, more in-depth clinical or pathophysiological research is required to firmly establish a connection between the two conditions.

Our findings showed that CRD is indeed an independent risk factor of HSK development (adjusted OR = 1.54, 95% CI = 1.39–1.70, *P* < 0.0001, Table 2). Few studies have explicitly explored the association between HSK and CRD. Some studies have examined HSV infection rates in dialysis patients and found a higher seroprevalence of HSV, although these differences were not statistically significant (Kao et al., 2002; Vilibic-Cavlek et al., 2017). We aimed to discuss the potential association between HSK and CRD from the perspectives of systemic inflammation and immune dysregulation. Research indicates that chronic HSV infections, including HSK, may exacerbate systemic inflammation (Kwon et al., 2018; Lobo et al., 2019; Musa et al., 2024), which is also a critical factor in CRD. Hypercytokinemia, a common feature of end-stage renal disease, may result from the accumulation of proinflammatory cytokines due to reduced renal clearance or increased production induced by uremic toxins, volume overload, or oxidative stress (Kimmel et al., 1998; Stenvinkel et al., 2005). This association may be linked to CRD-related changes in the immune system, potentially leading to decreased immune surveillance and increased susceptibility to HSV reactivation. This correlation highlights the need for nephrologists to be particularly attentive to patients with CRD and consider timely referrals for ophthalmic evaluation.

We found that, compared to the control group, patients with HIV infection had a significantly higher risk of developing HSK (adjusted OR = 2.60, 95% CI = 1.36–4.97, *P* = 0.0038). The association between HSK and HIV infection is well documented, with studies showing that HIV-positive individuals are at a markedly increased risk of developing severe and recurrent forms of HSK (Pramod et al., 2000; Burcea et al., 2015; Sobol et al., 2016). This heightened risk is largely attributed to immunosuppression caused by HIV, which compromises the body's ability to effectively control HSV infection. Regular ophthalmic assessments are crucial for monitoring and addressing these complications in HIV-infected patients.

Our study has several strengths. This is the largest investigation to date that focuses on patients with HSK, identifying 27,651 cases using the NHIRD database. Unlike studies that rely on patient self-reporting, our use of electronically recorded claims data minimized recall bias. Additionally, the nationwide scope of the NHIRD dataset eliminated selection bias related to referral centers. This case-control study also benefitted from a decade of longitudinal data on various sociodemographic factors and comorbidities in both patients and controls. Importantly, we considered these sociodemographic factors and comorbidities as potential confounding variables when evaluating the odds ratio for HSK.

This study had several limitations. Firstly, the identification of HSK cases and the absence of HSK in controls were based solely on claims data, not confirmed clinical records, which might lead to misclassification. Moreover, relying on ICD-9-CM codes for diagnosing HSK and comorbidities can also result in misclassification. As this is an observational study, causality cannot be established, and unmeasured confounders could skew the results. The findings may not be generalizable outside of Taiwan or to different healthcare systems. Furthermore, incomplete historical data and the lack of prior diagnoses verification for controls might affect the validity of the results. Additionally, the model's limited predictive accuracy, indicated by an AUC value of 0.5940, suggests that future research should include more relevant predictors to enhance model performance.

In summary, this study identified several sociodemographic factors associated with an increased risk of developing HSK, including living in Northern Taiwan and its metropolitan cities. After accounting for sociodemographic factors and potential comorbidities, our findings highlighted a significantly higher risk of developing HSK in patients with corneal abrasion, hyperlipidemia, DM, CAD, CRD, or HIV infection. Future studies should clarify these associations to enhance our understanding of the epidemiology and pathophysiology of HSK.

## Data Availability

The original contributions presented in the study are included in the article/supplementary material, further inquiries can be directed to the corresponding author.
